# New mouse model for inducible hACE2 expression enables to dissect SARS-CoV-2 pathology beyond the respiratory system

**DOI:** 10.1007/s00335-025-10115-1

**Published:** 2025-02-22

**Authors:** Federica Gambini, Dominik Arbon, Petr Nickl, Vaclav Zatecka, Olha Fedosieieva, Juraj Labaj, Vendula Novosadova, Jana Trylcova, Jan Weber, Jan Prochazka, Jana Balounova, Radislav Sedlacek

**Affiliations:** 1 Laboratory of Transgenic Models of Diseases, Institute of Molecular Genetics of the Czech Academy of Sciences, Videnska 1083, Prague, 142 20 Czech Republic; 2 Czech Centre for Phenogenomics, Institute of Molecular Genetics of the Czech Academy of Sciences, Prumyslova 595, Vestec, 252 50 Czech Republic; 3 Institute of Organic Chemistry and Biochemistry of the Czech Academy of Sciences, Prague, 166 10 Czech Republic

**Keywords:** SARS-CoV-2, Conditional mouse model, hACE2, Infection

## Abstract

The Severe Acute Respiratory Syndrome Coronavirus-2 (SARS-CoV-2) infection is not limited to the respiratory tract as receptors, including the angiotensin-converting enzyme 2 (ACE2), are expressed across many tissues. This study employed a new conditional mouse model, *Rosa26*^*creERT2/chACE2*^, which expresses human ACE2 (hACE2) across multiple organs, to investigate the effects of SARS-CoV-2 infection beyond the respiratory system. This strain demonstrated susceptibility to SARS-CoV-2 infection in a dose and sex-dependent manner, showing that infected male mice exhibited more severe disease outcomes, including significant weight loss, pronounced lung pathology and dysfunction, and increased mortality, compared to females. In contrast to intratracheal infection, intranasal virus administration facilitated viral spread to the brain, thereby underscoring the nasal route’s role in the pathogenesis of neurological manifestations. Intranasal infection also led to increased innate immune system activation as compared to intratracheal virus administration, even though both routes activated the adaptive immune response. This model provides a valuable tool to study SARS-CoV-2 in individual tissues or use a multisystemic approach, and it also advances possibilities for preclinical evaluation of antiviral therapies and vaccine strategies.

**Supplementary Information:**

The online version contains supplementary material available at 10.1007/s00335-025-10115-1.

## Introduction

Severe Acute Respiratory Coronavirus-2 (SARS-CoV-2), a novel coronavirus, emerged in late 2019 in Wuhan, China, causing the COVID-19 pandemic, which has since led to a global public health crisis. SARS-CoV-2 primarily infects the respiratory system, causing conditions ranging from mild respiratory symptoms to severe pneumonia and acute respiratory distress syndrome (ARDS) (Xiao et al. 2020). However, growing evidence has shown that the virus spread is not limited to the respiratory tract. The human angiotensin-converting enzyme 2 (hACE2) receptor, which was identified as the main receptor of viral entry, is not only present in the respiratory tract, but it is abundant in human small intestine, testis, kidney, heart, thyroid and adipose tissue (Li et al. 2020). Additionally, high protein levels of the receptor were found in the colon, rectum, adrenal gland, seminal vesicle, and gallbladder (HPA; Karlsson et al. 2021). Regarding the cell type, hACE2 was also shown to be abundantly present in human epithelial cells of the lung, enterocytes of the small intestine, and in endothelial cells of the arterial and venous vessels (Guney and Akar 2021). While hACE2 is essential for viral entry, SARS-CoV-2 infection also relies on host proteases to mediate cell invasion through two distinct pathways. The TMPRSS2-dependent membrane fusion pathway is the primary and more efficient route in TMPRSS2-expressing cells, facilitating direct fusion with the plasma membrane following spike protein cleavage at the S2’ site. Alternatively, in TMPRSS2-deficient environments, SARS-CoV-2 utilizes the cathepsin-dependent endocytic pathway, which enables viral uptake through endocytosis and subsequent spike activation within the endosomal compartment. The balance between these pathways varies across cell types and viral variants, influencing infectivity, tissue tropism, and disease progression (Iwata-Yoshikawa, 2022). In the context of the wide and abundant expression of the hACE2 as a main entry receptor, SARS-CoV-2 is now considered to be a multisystemic pathogen, that may cause post-acute sequelae or a post-acute syndrome (Cai et al. 2024) after the acute phase syndromes, all which may affect various organs. The post-acute syndrome, the long-COVID, may harm many organs, having long-term and even more devastating effects than the syndromes of the acute period, including heart thrombosis leading to myocardial infarction, inflammation, lung fibrosis, stroke, venous and arterial thromboembolism, brain fog, general mood dysfunctions, dermatological issues, persistent fatigue, and others (Sanyaolu et al. 2022).

Indeed, it has been shown that the virus can cause strong extrapulmonary manifestations through direct cytopathic effects and systemic inflammation (Temgoua et al. 2020) and the patients can develop cardiovascular complications such as myocarditis and acute heart failure, or severe kidney injury linked to cytokine storm (Spuntarelli et al. 2020; Zheng et al. 2021). Neurological manifestations are also significant, including headache, loss of smell and taste, and cognitive disturbances such as confusion and brain fog (Ding and Zhao 2023; Gupta et al. 2020). Systemic effects of the virus have been further emphasized by haematological abnormalities, including impairment in the coagulation system, resulting in an increased tendency of thrombosis. In severe cases of SARS-CoV-2 infection, multisystem inflammatory syndrome may occur, also known as MIS-C in children and MIS-A in adults. These disorders are defined by high fever, widespread inflammation, and multi-organ dysfunction, including the heart, lungs, or kidneys (Gupta et al. 2020).

The multisystem nature of COVID-19 has highlighted the importance of animal models to study viral behaviour, host responses, and potential treatments. The development of genetically modified mice expressing the hACE2 receptor, had enabled researchers to study viral behaviour and host responses through in vivo experiments.

SARS-CoV-2 exhibits a negligible affinity to murine ACE2 in comparison to human ACE2, therefore the mouse ACE2 receptor does not effectively support SARS-CoV-2 entry to the mouse host. To study the biological aspects of SARS-CoV-2, the development of humanized mouse models needs to be undertaken (Nickl et al. 2022), especially to understand the biology and pathology in multiple organs.

The COVID-19 pandemic underscored the urgent need for animal models that would facilitate rapid investigation of SARS-CoV-2 and enable testing of potential therapeutics to mitigate systemic disease. In response, the scientific community prioritized available transgenic models, such as the K18-ACE2 mouse (McCray et al. 2007) and the HFH4-ACE2 mouse (Menachery et al. 2016), which drive hACE2 expression primarily in epithelial cells and lungs, respectively. However, these models provided only a partial understanding of SARS-CoV-2 infection due to tissue-specific promoter-driven expression of the receptor.

While these models have primarily been employed to investigate the effects of the virus on the respiratory system, growing evidence highlights the viral impact beyond the lungs, necessitating models that enable the study of systemic organ involvement. To address this, we developed and characterized a conditional mouse model, in which the human ACE2 receptor can be expressed in a controlled, tissue-specific, or time-dependent manner. This can be achieved using a tissue-specific Cre-driver mouse strain or tamoxifen inducible Cre-driver to allow precise spatiotemporal regulation of hACE2 expression. We describe the generation and characterization of this conditional model, which facilitates the studies of SARS-CoV-2 infection beyond the respiratory system and can provide tools for more comprehensive understanding the systemic consequences of COVID-19.

## Materials and methods

### Production of *Rosa26*^*creERT2/chACE2*^ transgenic mice

**Fig. 1:**
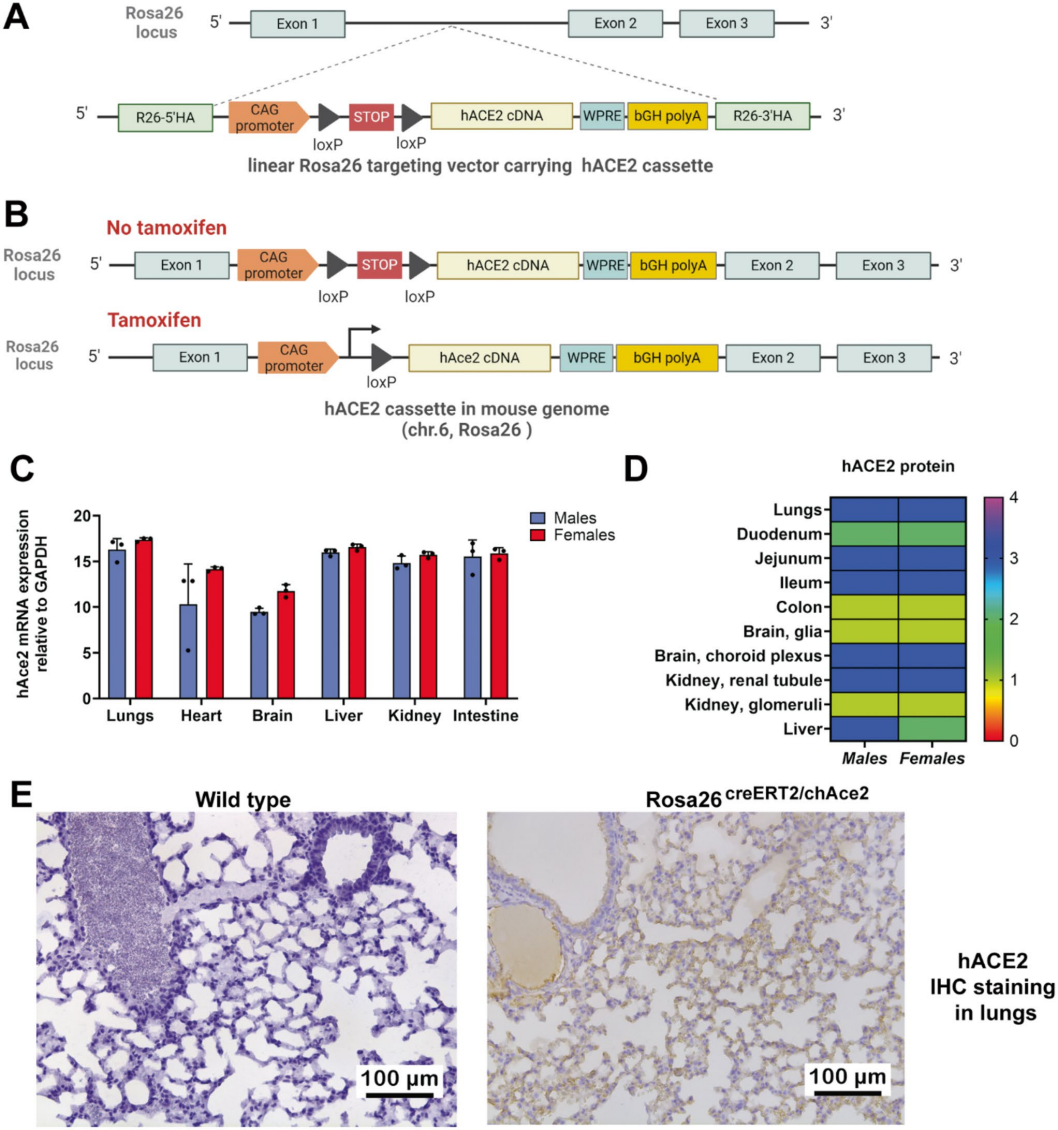
Rosa26^creERT2/hACE2^ mouse model generation and hAce2 characterization. **(A**) Conditional CAG-loxP-STOP (3xSV40 polyA)-loxP-hACE2(CDS) cassette was inserted into the mouse Rosa26 (chr.6) locus using CRISPR/Cas9 system. CAG = **C**-Cytomegalovirus (CMV) early enhancer, **A**- chicken β-Actin promoter + rabbit β-**G** lobin chimeric intron. WPRE = Woodchuck Hepatitis Virus **P**osttranscriptional**R**egulatory**E**lement was included in the construct to enhance mRNA stability and increase expression levels of the transgene, as demonstrated in previous studies utilizing the Rosa26 locus (Madisen et al., 2010). bGH polyA = **b**ovine**G**rowth**H**ormone polyadenylation signal. (**B**) Tamoxifen-induced Cre recombination excises the STOP cassette, allowing hACE2 expression. (**C**)– Quantification of relative mRNA expression of hACE2 in different organs of Rosa26^creERT2/chAce2^ transgenic mice after tamoxifen treatment. Data are shown as mean ± SD for male and female mice. n = 3 for each group. (**D**) Quantification of hACE2 protein levels and distribution across tissues of Rosa26^creERT2/chAce2^ males and females based on hACE2 IHC staining (0- none, 1 - low, 2 - moderate, 3 - strong, 4– very strong.) Data are shown as mean. n = 5 for males, n = 6 for females. (**E**) Example IHC staining of hACE2 protein in the lungs of wild type and Rosa26^creERT2/chAce2^ mice after tamoxifen administration

*The Gt(ROSA)26Sor*^*em1(ACE2)Ccpcz*^ mouse line was created by insertion of a conditional cassette of HA-CAG-loxP-stop (3xSV40 polyA)-loxP-hACE2(cDNA)-HA into the *Rosa26* locus through pronuclear microinjection into C57Bl/6NCrl zygotes, using the gRNA sequence 5’-CTCCAGTCTTTCTAGAAGAT. The cassette insertion was verified through genotyping and sequencing at the 5′ end using the primers 5′F-GCACTTGCTCTCCCAAAGTC and 5′R-GGGCGTACTTGGCATATGAT. Genotyping and sequencing of hACE2 to check the integrity of the sequence were performed using primers 5′F-AGAACCCTGGACCCTAGCAT and 3′R-TGATGGCCTTTTCAACTTCA. The insertion of the cassette at the 3′ end into the genome was demonstrated using primers 5′F-TAGTTGCCAGCCATCTGTTG and 3′R-GCCAGTCCAAGAGAAAGCAC. *Gt(ROSA)26Sor*^*em1(ACE2)Ccpcz*^ mice were crossed to homozygotes and further bred with *B6.129-Gt(ROSA)26Sor*^*tm1(cre/ERT2)Tyj/*^*J* mice (The Jackson Laboratory strain 008463), common name R26-CreERT2 (https://www.jax.org/strain/008463). The offsprings were genotyped for the presence of both hACE2 and R26-CreERT2 constructs using the mentioned primers and protocol provided by the Jackson Laboratory, respectively. In this work, we refer to *Gt(ROSA)26Sor*^*em1(ACE2)Ccpcz*^ / B6.129-Gt(ROSA)26Sor^tm1(cre/ERT2)Tyj/^J mice as *Rosa26*^*creERT2/chACE2*^. All mice were administered tamoxifen intraperitoneally (2.7 mg, three doses, 24 h in between) at least three days before the experiment to ensure hACE2 expression (Fig. 1).

### SARS‑CoV‑2 infection

Mice were anesthetized using Isoflurane and immediately infected intranasally with 1 × 10^4^ PFU of virus in 50 µl. The infection was performed with SARS-CoV-2 virus, strain B.1, isolate hCoV-19/Czech Republic/NRL_6632_2/2020, diluted in Dulbecco’s Modified Eagle’s Medium (DMEM) with no FBS. The infection was allowed to progress until the mice reached criteria for humane euthanasia. Mice were monitored daily for clinical signs of disease progression. In this study, animals were euthanized immediately upon reaching a 25% body weight loss to minimize suffering. Additional humane endpoints included persistent tachypnoea, significantly reduced mobility (including ataxia), dermatological manifestations of infection, roughened fur, and other indicators of deteriorating health. Signs such as hunched posture, repetitive behaviours, or prolonged inactivity (e.g., remaining in a corner of the cage without response to stimuli) were also considered. Animals meeting any of these criteria were humanely euthanized to prevent undue distress. Uninfected controls were administered DMEM. To analyse the influence of viral load on infection progression, different concentrations were tested, and responses were compared across groups. The virus was administered intranasally using 4 × 10^4^, 1 × 10^4^, 5 × 10^3^, 2.5 × 10³ and 1 × 10³ PFU in total volume of 50 µl. To analyse the influence of the route of virus administration, intratracheal infection of the same dose and volume of the virus was performed in parallel to the intranasal one. In the intratracheal infection procedure, mice were intraperitoneally anesthetized with Ketamin-Xylazin (Zolazepam, Tiletamine), intubated by cannula and after verification of correct intubation using FlexiVent system (Emka technologies), the virus was administered directly to the lungs, thereby by-passing its passage through the nasal cavity. Mice were monitored until regaining full consciousness.

### Plaque forming assay (PFA)

Tissues, including lung, brain, heart, liver, kidney, and small intestine were collected from mice at either 2-, 5-, and 7-days post-infection (dpi). Then, each tissue was weighed and homogenized in phosphate-buffered saline (PBS) to a 20% suspension, using a TissueLyser III (Qiagen), 5 min, 30 Hz. Debris was separated by centrifugation (10 000 g, 10 min, 4 °C) and discarded. Viral titration was performed using VERO E6 cells. The cell monolayer was gently washed with PBS and the cleared tissue homogenates were added to the cells in 10× series dilutions and incubated at 37 °C for one hour with regular shaking. Each well was overlayed with 500 µl of 1.5% carboxymethyl cellulose in DMEM and the cells were incubated for 72 h at 37 °C, 5% CO_2_. Cells were then washed with PBS and stained with crystal violet solution in acetic acid for 15 min to visualize the plaques. Before scoring a final PBS wash was performed to ensure clear visualization of the plaques.

### RNA extraction from tissues and cDNA conversion

Tissues were collected from both infected mice and uninfected controls on 2, 5, and 7 dpi. Wild-type mice were used as negative controls to establish the baseline levels for mouse *Ace2* expression. Total RNA was extracted using Trizol reagent (Thermo Fisher Scientific) and homogenized with TissueLyser III (Qiagen), 5 min, 30 Hz. 2 µg of RNA was converted into cDNA. The cDNA conversion was performed by using M-MLV Reverse Transcriptase (Promega, M170B) and its M-MLV RT 5X Buffer (Promega, M531A), together with rRNAsin^®^ (Promega, N251B) for RNAse activity inhibition. After overnight incubation at 37 °C, the samples were diluted with 100 µl of RNAse-free water.

### Real time qPCR

Real-time qPCR was performed on LightCycler instrument by Roche, using LightCycler 480 SYBR Green I Master (Roche, Cat. No 04887352001, Germany) with *Gapdh* as internal control. Three sets of primers were used for the detection of viral mRNA, targeting the mRNA of Nucleocapsid (NP), Spike, and RNA-dependent RNA polymerase (RdRP). Primer sequences targeting Nucleocapsid mRNA have been obtained from Wozniak and Cerda et al. 2020 (Wozniak et al. 2020), and Spike and RdRP primer sequences were taken from a study by Park and Won et al. 2020 (Park et al. 2020). One set of primers was used to detect h*ACE2* mRNA. All primer sequences are listed in Table 1.

**Table 1:** Sequences of qPCR primers used for the quantification of viral gene, hACE2 and gapdh mRNA

NP	TTACAAACATTGGCCGCAAA
	GCG CGA CAT TCC GAAGAA
Spike	GCTGGTGCTGCAGCTTATTA
	AGGGTCAAGTGCACAGTCTA
RdRP	AGAATAGAGCTCGCACCGTA
	CTCCTCTAGTGGCGGCTATT
h*ACE2*	TCCATTGGTCTTCTGTCACCCG
	AGACCATCCACCTCCACTTCTC
*Gapdh*	CGTCCCGTAGACAAAATGGT
	TTGATGGCAACAATCTCCAC

### Flow cytometry analysis

Blood samples were collected from mice under Isofluorane anaesthesia, from retro-orbital venous sinus, prior to SARS-CoV-2 infection, and at 2, 5, or 7 dpi. Within the BSL-3 facility, 50 µl of blood was stained with 50 µl of antibody cocktail (Online resource 1) with eBioscience™ Fixable Viability Dye eFluor™ 455UV (ThermoFisher Scientific) and mouse Fc block (BD Biosciences) followed by the Lyse/Wash procedure with BD FACS™ lysing buffer (BD Biosciences). To ensure complete virus inactivation, samples were treated with 1.6× BD FACS lysis solution for at least 15 min before their transfer to BSL2 facility (Eddins et al. 2022). Samples were then washed with 2 ml FACS buffer (2% FCS, 2mM EDTA in HBSS w/o Mg^2+^, Cl^2+^) and acquired on Cytek Aurora Full Spectrum Flow Cytometer (Cytek Biosciences). Data was analysed in FlowJO software (BD Biosciences). The gating strategy is shown in Online resource 2.

### Lung function

Before the infection and on 7 dpi, mice were anesthetised by intraperitoneal administration of Ketamin-Xylazin (Zolazepam, Tiletamine), intubated and lung function parameters were measured using FlexiVent (Emka technologies). Resulting data was relativized to the current weight of the animals. Outliers were excluded using Grubbs test and evaluated by two-way ANOVA.

### Histopathology analysis

Organs were collected (lung, brain, heart, liver, kidney and intestine) and fixed in 4% formaldehyde. After fixation, samples were transferred to 70% ethanol. The samples were processed by the Leica ASP6025 Vacuum Tissue Processor according to the program Standard processing overnight, embedded in Paraplast X-tra (Leica Biosystems), using the Tissue Embedding Station Leica EG1150. The Sect. (2 μm thick) were prepared using the Leica Fully Motorized Rotary Microtome RM2255-FU. The obtained slides were stained by Hematoxylin H (Biognost) and Eosin Y (Carl Roth) using the Leica ST5010-CV5030 Stainer Integrated Workstation. Sections were imaged using Microscope Leica DM 3000 LED (100x magnification) equipped with Leica DFC450 colour camera. Images were analysed in Zeiss Zen 3.9 software.

### Immunohistochemistry (IHC)

Organs were collected and processed as reported in the paragraph “Histopathology analysis”. IHC was performed to determine the presence of the human ACE2. Paraffin-embedded tissue sections were deparaffinized and antigen retrieval was performed at pH9. Endogenous peroxidase activity was blocked, followed by incubation with 2% bovine serum albumin (BSA) for 30 min at room temperature (RT) to reduce non-specific binding. The sections were then incubated with the primary antibody against hACE2 (*ab108209*, Abcam), at a dilution of 1:500 overnight at RT. After washing, sections were incubated with ZytoChem Plus-HRP Polymer anti-Rabbit antibody (ZUC032-100, Zytomed Systems GmbH) 30 min at RT and developed with a DAB chromogenic reaction (K3468, Dako). Counterstaining by Harris Hematoxylin (HEMH-OT-2.5 L, Baria) and mounting of slides was done automatically by Leica ST5020 + CV5030 stainer and coverslipper. Sections were imaged using Microscope Leica DM 3000 LED (100x magnification) equipped with Leica DFC450 colour camera. Images were analysed in Zeiss Zen 3.9 software. IHC for hACE2 was quantified using an internal semi-quantitative scoring system based on expression intensity and distribution within each organ. Expression was graded on a five-point scale: 0- None, 1 - Low, 2 - Moderate, 3 - Strong, 4– Very strong. For each organ, both the intensity of hACE2 expression and the degree of distribution across the tissue were assessed.

### Statistical analysis

Data were analysed using a linear mixed model with the formula variable ~ Treatment * Sex, including animal as a random effect. Contrasts were designed for subsequent comparisons. All analyses were performed in R (version 4.4.2) using the packages *lmer* and *multcomp* (*glht* function).

### Study approval

This study complied with Czech national laws on animal experimentation and cruelty prevention (Animal Welfare Act No. 246/1992 Coll.). The protocol received approval from the Ethics Committee of the Institute of Molecular Genetics, Czech Academy of Sciences of the Czech Republic (permit AVCR 10175/2022 SOV II). All work with SARS-CoV-2 virus and infectious material was performed in a certified BSL3 laboratory at the Czech Centre for Phenogenomics, hosted by Institute of Molecular Genetics of the Czech Academy of Sciences.

## Results

### *Rosa26*^*creERT2/chACE2*^ transgenic mice express human ACE2, the primary receptor for SARS-CoV-2 entry

We have used *Rosa26*^*creERT2/hACE2*^ mice to ensure a widespread expression of hACE2 upon tamoxifen administration (Fig. 1A-C). Expression of human hACE2 was verified both at the RNA and protein level showing major expression in the lungs, brain, heart, liver, kidneys, and intestine (Fig. 1C-E). Tamoxifen induction activated hACE2 expression, resulting in increased mRNA and protein levels following induction in *Rosa26*^*creERT2/chAce2*^ mice (Fig. 1C-E).

### The virus titer and sex modulate the disease severity upon intranasal SARS-CoV-2 infection

**Fig. 2:**
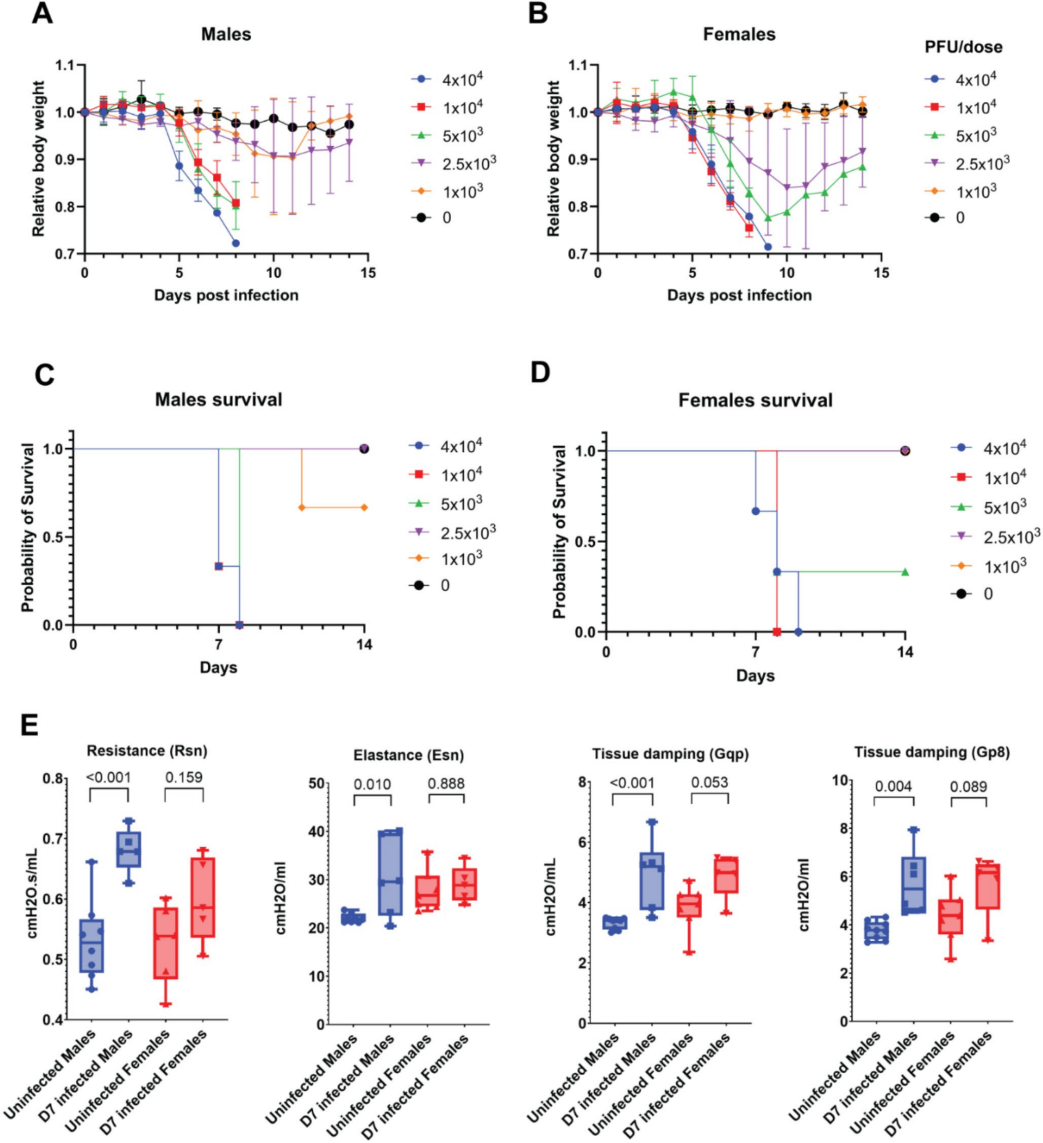
Rosa26^creERT2/chAce2^ mice are susceptible to SARS-CoV-2 infection, affecting the lung function. (**A**) Dose-dependent body weight loss in Rosa^26creERT2/chAce2^ males infected with increasing concentrations of SARS-CoV-2 (PBS, 1 × 10³, 2.5 × 10³, 5 × 10³, 1 × 10^4^, and 4 × 10^4^ PFU per dose). Males showed significant body weight loss compared to their initial body weight at viral concentrations of 5 × 10³ PFU/dose and above, with the highest concentration (1–4 × 10^4^ PFU/ dose) resulting in mortality. Data are shown as relative body weight ± SD. *n* = 3 for each dose. (**B**) Rosa^26creERT2/chAce2^ females infected with same increasing concentrations of SARS-CoV-2 exhibited a similar pattern, with more severe infection outcome at higher viral load. Females infected with 2.5 × 10³ and 5 × 10³ PFU/ dose experienced severe body weight loss but eventually recovered. Data are shown as relative body weight ± SD. *n* = 3 for each dose. (**C**) Survival curve of Rosa^26creERT2/chAce2^ males infected with increasing concentrations of SARS-CoV-2. (**D**) Survival curve of Rosa^26creERT2/chAce2^ females infected with increasing concentrations of SARS-CoV-2. (**E**) FlexiVent analysis showed impaired lung functionality in infected males compared to uninfected mice and infected females. Resistance (Rsn), elastance (Esn) and tissue damping (Gqp, Gp8)

The *Rosa26*^*creERT2/chAce2*^ mice are susceptible to SARS-CoV-2 B.1 variant responsible for the first pandemic wave, infection in a dose dependent manner as can be inferred from application of five different virus concentrations (1 × 10³, 2.5 × 10³, 5 × 10³, 1 × 10^4^, and 4 × 10^4^ PFU/ dose) (Fig. 2A-B). Mice mock-infected with PBS did not show any body weight loss. High viral loads (1 × 10^4^, and 4 × 10^4^ PFU) led to a fatal infection in all mice around day 8 post infection (dpi). At viral titres of 1 × 10³ to 5 × 10³ PFU/ dose, at least some mice showed longer survival with signs of body-weight recovery (Fig. 2C-D). Female mice generally exhibited less lethality, while males succumbed to the disease. These results revealed a consistent trend, highlighting the critical role of viral load and sex in determining the outcome of SARS-CoV-2 infection in *Rosa26*^*creERT2/chAce2*^ mice, leading to our decision to use 1 × 10^4^ PFU/ dose for follow up studies. Similarly to our previous study utilizing AAV-ACE2-humanized mouse model (De Gasparo et al. 2021). This dose caused progressive weight loss, respiratory pathology, including alveolar wall destruction, thickening of septa, immune cell infiltration and terminal disease by 8 dpi (Fig. 2A-B and Online resource 3A).

### Lung function in males infected with SARS-CoV-2 was decreased in comparison to females

The lung tests revealed that males intranasally infected with SARS-CoV-2 showed a statistically significant increase in lung resistance (Rsn) at 7 dpi (Fig. 2E). This data suggests that the infection leads to obstruction, likely due to inflammation. Infected males also showed an increase in elastance (Esn) and increase tissue damping (Gqp, Gp8) (Fig. 2E). These parameters are a measure of dissipated energy in lung tissue due to the stiffness and resistance of peripheral airways and lung parenchyma (Winkler et al. 2020). Although infected females did not show significant changes in lung function parameters, there was a slight increase in tissue damping parameters, which is consistent with the milder disease severity in females. On the contrary, the lung capacity (Lc) remained unchanged between groups (*p* = 0.932 for males, *p* = 0.997 for females). Similarly, other lung function parameters—including dynamic compliance (Csn), Newtonian resistance (Rqp, Rp8), and tissue elastance (Hqp, Hp8)—showed no significant differences. The only exception was Newtonian resistance (Rp8) in males, which showed a slight increase post-infection (*p* = 0.013) (Online resource 3B).

### Intranasal and not intratracheal infection leads to severe COVID-19 disease in *Rosa26*^*creERT2/chACE2*^ mice

**Fig. 3:**
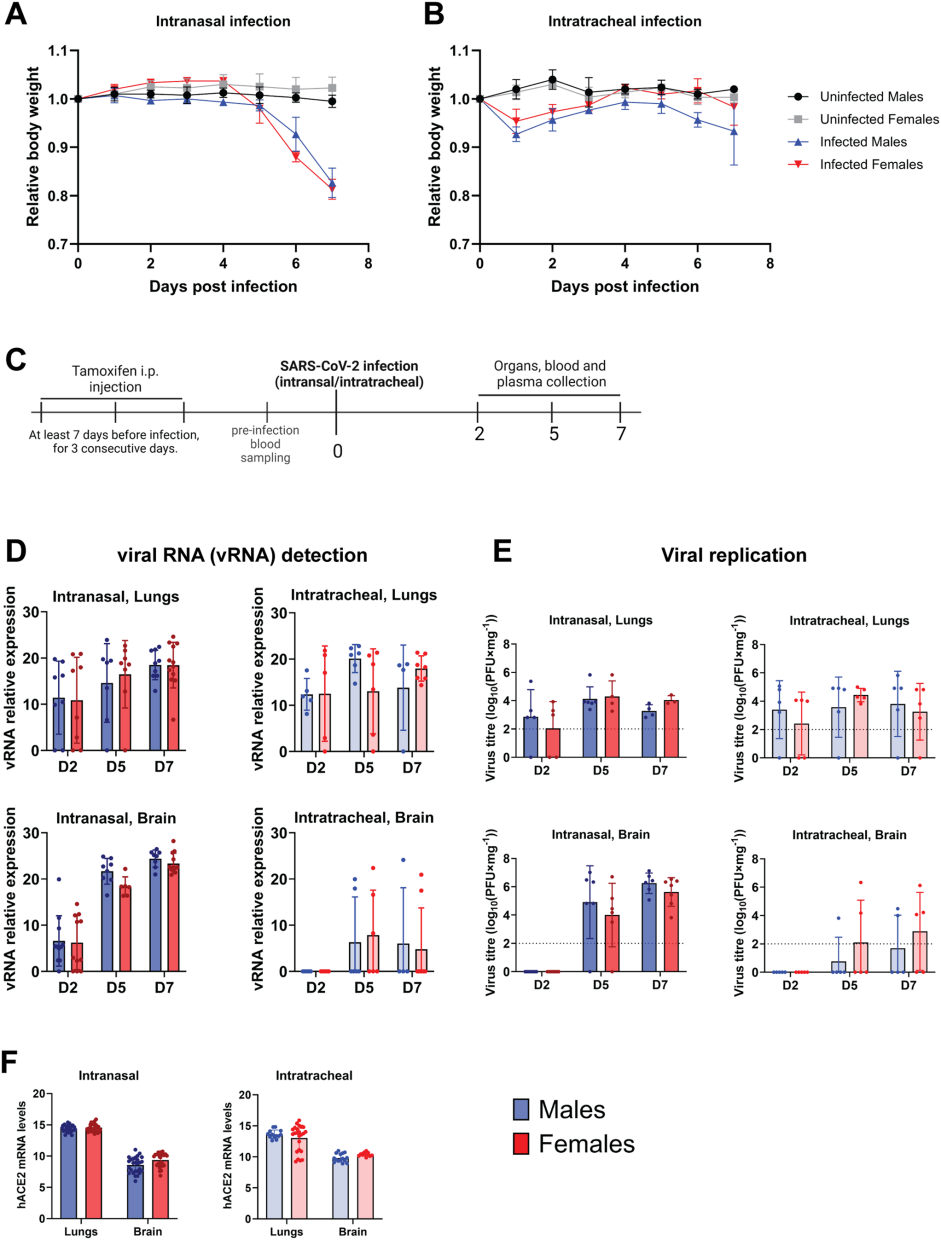
Intranasal infection facilitates the virus spread to the brain in Rosa26^creERT2/chAce2^ mice. (**A-B**) Body weight curves during intranasal and intratracheal infections. (**A**) Intranasal infection resulted in a noticeable decrease in body weight, which correlates with clinical signs of infection, including hunched posture, reduced activity, and general malaise. (**B**) In contrast, intratracheal infection did not lead to a significant reduction in body weight or the visible onset of clinical symptoms. Data are shown as relative body weight ± SD. (**C**) Experimental scheme for SARS-CoV-2 infection model. Mice were administered tamoxifen via intraperitoneal (i.p.) injection at the indicated time point prior to infection. SARS-CoV-2 infection was performed either intranasally or intratracheally on day 0. Following infection, samples of blood, plasma, and organs were collected at the specified time points for downstream analysis. (**D**) Viral RNA (v RNA) detection in the lungs or brain of infected Rosa26^creERT2/chAce2^ mice. Animals infected intratracheally showed a lower presence of the virus in the brain compared to the ones infected intranasally. Data are shown as mean ± SD. For Lungs - Intranasal: D2 (*n* = 8 males, *n* = 8 females); D5 (*n* = 7 males, *n* = 8 females); D7 (*n* = 9 males, *n* = 12 females). Intratracheal: D5 (*n* = 5 males, *n* = 5 females) and (*n* = 6 males, *n* = 6 females); D7 (*n* = 8 males, *n* = 7 females). For Brain - Intranasal: D2 (*n* = 11 males, *n* = 11 females); D5 (*n* = 8 males, *n* = 6 females); D7 (*n* = 8 males, *n* = 12 females). Intratracheal: D2 (*n* = 11 males, *n* = 11 females); D5 (*n* = 8 males, *n* = 6 females); D7 (*n* = 8 males, *n* = 12 females). (**E**) Plaque forming assay (PFA) showing SARS-CoV-2 replication in the lungs and brain of infected mice. Results were consistent with viral RNA detection. Data are shown as mean ± SD For Lungs - Intranasal: D2 (*n* = 5 males, *n* = 5 females); D5 (*n* = 6 males, *n* = 4 females); D7 (*n* = 4 males, *n* = 3 females). Intratracheal: *n* = 5 males, *n* = 5 females for all timepoints. For Brain - Intranasal: D2 (*n* = 7 males, *n* = 7 females); D5 (*n* = 6 males, *n* = 6 females); D7 (*n* = 6 males, *n* = 7 females). Intratracheal: *n* = 5 males, *n* = 5 females for all timepoints. (**F**) hACE2 mRNA levels in intranasally and intratracheally infected mice. Data are shown as mean ± SD

To study the impact of the infection route, mice were infected with SARS-CoV-2 intranasally or intratracheally. Their body weight was assessed daily for a period of 7 dpi to assess the virus spread, replication and disease progression. Intranasally infected mice were highly susceptible to the virus, as shown by the body weight loss in Fig. 3A. Around 5 dpi, infected mice began exhibiting clinical symptoms, including hunched posture, reduced activity, and occasional signs of malaise, which coincided with a significant drop in body weight. As the disease progressed, these symptoms became evident, leading to death or humane euthanasia by 7 dpi. Notably, males displayed these symptoms earlier and more severely than females, as reflected in the survival curves (Fig. 2C-D), indicating a higher disease severity in males compared to females. In contrast, intratracheally infected animals did not show significant body weight drop or any other symptoms associated with the infection (Fig. 3B).

### Intranasal infection facilitates the virus spread to the brain

We proceeded with studying the dynamic of the infection at three different timepoints (2, 5, and 7 dpi) in the lung, brain, heart, liver, kidney, and intestine, separately for infected females and infected males due to their different sex-specific disease outcome. The timepoints were chosen based on the progression of the body weight loss, reflecting the disease severity development. Intranasally infected *Rosa26*^*creERT2/chACE2*^ mice did not show any visible symptoms at 2 dpi. However, at 5 dpi, they started to lose weight significantly and at 7 dpi they had to be terminated due to their critical condition (Fig. 3C). The presence of SARS-CoV-2 was assayed by qPCR using primers against three viral genes: the two structural proteins, Spike and Nucleocapsid (NP), and the non-structural protein RNA-dependent RNA polymerase (RdRP). Viral RNA was detected in the lungs and brain of intranasally infected *Rosa26*^*creERT2/chACE2*^ mice, while no virus was detected in the rest of the organs (data not shown). In the lungs, viral RNA was detected as early as at 2 dpi in the majority of mice (both males and females), with a consistently high viral load observed during the course of infection at following time points. This pattern was consistent in both intranasally and intratracheally infected mice. Of note, some mice showed no detectable virus at 2 dpi, likely due to the viral load being below the detection threshold. In contrast, the viral load increased gradually in brains of intranasally infected animals from 2 dpi, peaking at 7 dpi (Fig. 3D). While upon intranasal infection the virus replicated in the brain already at 2 dpi, no viral RNA as well as no replication was detected in the brains of intratracheally infected mice at 2 dpi. Moreover, only a fraction of intratracheally-infected animals showed viral RNA presence or replication at 5–7 dpi. This might explain the milder outcome of the infection in intratracheally-infected mice (Fig. 3D-E). To demonstrate that the differences in virus spread were not caused by differences of hACE2 expression among individual tissues and animals, *hACE2* mRNA expression was quantified during the infection in the infected organs, and no significant change was detected in either intranasal or intratracheal infection (Fig. 3F). Thus, the viral entry to the brain is facilitated by the intranasal virus application.

### Intranasally infected mice show a robust immune response to SARS-CoV-2

**Fig. 4:**
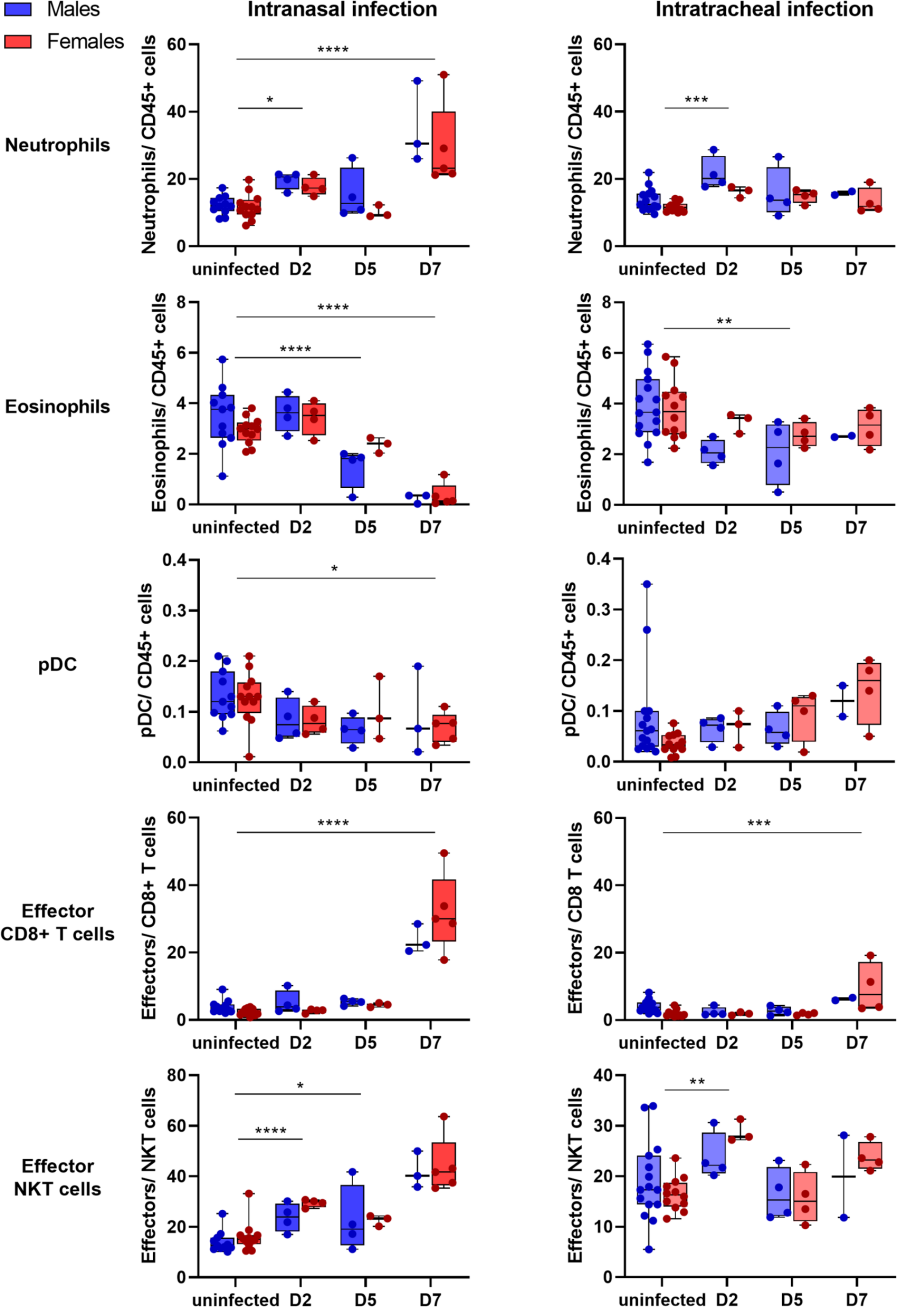
Immune response in Rosa26^creERT2/chAce2^ mice following infection via intranasal and intratracheal route. Data are shown as mean ± SD For intranasal infection: Uninfected (*n* = 11 males, *n* = 12 females), D2 (*n* = 4 males, *n* = 4 females), D5 (*n* = 4 males, *n* = 3 females), D7 (*n* = 3 males, *n* = 5 females). For intratracheal infection: Uninfected (*n* = 15 males, *n* = 12 females), D2 (*n* = 4 males, *n* = 3 females), D5 (*n* = 4 males, *n* = 4 females), D7 (*n* = 2 males, *n* = 4 females)

We have monitored the immune response in *Rosa26*^*creERT2/chAce2*^ mice during the infection in peripheral blood, following both intranasal and intratracheal SARS-CoV-2 infection (Fig. 4). Aligning with severe disease symptoms, intranasally infected *Rosa26*^*creERT2/chAce2*^ mice showed an increased percentage of neutrophils and decreased percentage of eosinophils, plasmacytoid dendritic cells (pDCs), B cells and Tregs in their peripheral blood (Fig. 4 and Online resource 4). Additionally, at 7 dpi, these mice showed a marked increase in effector cytotoxic (CD8+) T cells (9.3x) and effector NKT cells (2.9x). In contrast, intratracheally infected mice displayed a smaller increase in effector cytotoxic (CD8+) T cells (2.7x) at 7 dpi, and neutrophils, eosinophils, and pDCs remained relatively unchanged. This indicates that, while innate immune response was relatively mild, some adaptive immune response was also activated following intratracheal infection (Fig. 4 and Online resource 4). Of note, while monocytes were also slightly increased in the initial stages of infection, we have observed a shift from the classical (Ly6C+, CD43-) to intermediate (Ly6C + CD43+) and non-classical (Ly6Clow, CD43+) monocyte phenotypes over the course of intranasal infection (Meghraoui-Kheddar et al. 2020). We have observed a similar, however, less significant trend following the intratracheal infection (Online resource 5). In summary, the severe disease outcome associated with intranasal, but not intratracheal, infection correlated with an enhanced innate immune response, indicated by elevated circulating neutrophils, and a robust adaptive immune response, marked by increased effector CD8 + cytotoxic T cells, accompanied by reduced eosinophils, pDCs, Tregs, and B cells (Fig. 4 and Online resource 4).

## Discussion

In this work, we exemplify the usage of new mouse model conditionally expressing hACE2. We describe here how various dosages of the SARS-CoV-2 virus B.1 variant impact the infection outcome, including survival, virus spreading into several organs, lung function and immune responses in two application routes, the most common intranasal route and in the intratracheal route.

The intranasal infection resulted in a dose-dependent disease severity, with higher viral loads, leading to more severe weight loss and mortality, particularly in males. Viral dose titration experiments in intranasally infected mice have shown, that males are more sensitive to SARS-CoV-2 infection, which aligns with higher COVID-19 mortality in male patients (Peckham et al. 2020). Additionally, the intranasal infection of *Rosa26*^*creERT2/chAce2*^ mice, unlike intratracheal infection, facilitated faster viral spread to the brain, highlighting the role of the nasal route in neurological manifestations.

The susceptibility of mouse models to SARS-CoV-2 infection, at least in the original variant and its derivations, is generally assessed by dramatic decrease in body weight, with consecutive presence of the virus in lungs and the brain, which all correlates with disease severity. Elevated levels of infectious virus have been detected in the brains of several published COVID-19 mouse models (Kumari et al. 2021; Sun et al. 2020). In these models, viral presence in the brain is a key factor contributing to the infection’s fatal outcome. This is particularly evident in the widely used K18-hACE2 mouse model, where SARS-CoV-2 neuroinvasion appears to play a significant role in disease mortality (Usai et al. 2023). In contrast to the situation in infected mice, the central nervous system (CNS) is not typically considered a major site of infection in humans, although SARS-CoV-2 has been reported in some cases (Krasemann et al. 2022; Kujawska et al. 2023). This discrepancy underscores a limitation of current mouse models, which do not fully reflect neurotropism observed in human COVID-19 infections. When *Rosa26*^*creERT2/chACE2*^ mice are infected via the intratracheal route, the viral load in the brain is significantly lower compared to the intranasal route. The intratracheal infection delivers the virus directly to the lungs, bypassing the nasal passage and preventing interactions with the olfactory mucosa and neural-mucosal interface, delaying brain invasion and resulting in lower percentage of mice with detectable virus in the brain. In fact, it has been proposed that respiratory viruses, including SARS-CoV-2, can access the brain through several potential routes, such as hematogenous spread via infected immune cells, direct infection of endothelial cells and pericytes and the disruption of the blood-brain barrier (BBB) (Ahmed et al. 2023; Zhang et al. 2021). These findings suggest that adjusting the route of infection in animal models could provide a more accurate representation of human COVID-19 conditions.

SARS-CoV-2 infection is associated with neurological symptoms, including headache, dizziness, loss of taste and smell, cognitive impairments (commonly referred to as “brain fog”), anxiety, and depression (Boldrini et al. 2021; Chen et al. 2021). While these symptoms are most often linked to severe cases, mild cases have also been associated with minor cognitive deficits (Woo et al. 2020). This underscores the need for further investigation into how the virus spreads within the brain. Postmortem analyses of COVID-19 patients have detected SARS-CoV-2 particles and viral RNA in the olfactory mucosa and in neuroanatomical regions receiving projections from the olfactory tract, evoking the possibility that axonal transport at the neural-mucosal interface in the olfactory mucosa may be one of a key mechanism through which the virus enters the brain (Meinhardt et al. 2021). In humans, this can be attributed to the higher presence of transmembrane protein serine protease 2 (TMPRSS2) and ACE2 within the olfactory epithelium cells (Ahmed et al. 2023). Given the role of TMPRSS2 in facilitating SARS-CoV-2 entry, we hypothesize that the murine orthologue of TMPRSS2 functions analogously to its human counterpart in mediating viral entry into host cells. This hypothesis is supported by evidence demonstrating that TMPRSS2 proteins from genetically diverse species exhibit conservation of key protein domains necessary for viral spike S2’ cleavage (Qu et al. 2024). Furthermore, TMPRSS2-mediated viral entry enhancement has been observed across evolutionarily divergent mammalian orthologues, including murine TMPRSS2, in multiple SARS-CoV-2 variants (Qu et al. 2024). Notably, while TMPRSS2 facilitates more efficient viral entry, SARS-CoV-2 can still utilize an alternative cathepsin-dependent endocytic pathway in TMPRSS2-deficient environments (Iwata-Yoshikawa et al. 2022), suggesting a degree of flexibility in viral entry mechanisms.

In this complex scenario, further research is essential to fully understand how SARS-CoV-2 accesses the brain.

Intranasally infected *Rosa26*^*creERT2/chAce2*^ mice developed severe COVID-19 symptoms and mounted a robust immune response while intratracheally infected mice exhibited limited activation of the immune system and did not develop severe symptoms. This highlights the critical role of the nasal mucosa in the development of a pathogenic immune response. The increase in neutrophils in the peripheral blood suggests the activation of emergency granulopoiesis in response to SARS-CoV-2 infection. Paradoxically, eosinophil levels declined in the bloodstream over the course of infection, likely due to their migration to the infected organs. This is consistent with elevated G-CSF and eotaxin levels observed in the serum of aged SARS-CoV-2 infected hACE2 knock-in mice and in the lungs of K18-hACE2 mice (Sun et al. 2020; Winkler et al. 2020). Similarly to the findings in K18-hACE2 mice and COVID-19 patients, B cells were decreased upon SARS-CoV-2 infection in *Rosa26*^*creERT2/chAce2*^ mice (Winkler et al. 2020; Zhou et al. 2020). Notably, a recent study detected elevated eotaxin-1/CCL11 levels in the cerebrospinal fluid of SARS-CoV-2-infected AAV-hACE2 mice. And additionally, long-COVID patients with cognitive deficits were found to have increased plasma CCL11 levels (Fernández-Castañeda et al. 2022), suggesting that eosinophils and neutrophils recruited to infected lungs and the brain may contribute to localized pathologies.

Intranasally infected *Rosa26*^*creERT2/chACE2*^ mice also exhibited reduced pDCs in their peripheral blood, mirroring observations in COVID-19 patients (Hadjadj et al. 2020), which implies that this animal model could be valuable to study protective roles of pDCs against SARS-CoV-2 infection. In the *Rosa26*^*creERT2/chAce2*^ model, monocyte levels initially increased during intranasal infection. Over time, classical monocytes declined while intermediate and non-classical monocytes increased. A similar shift was observed in septic patients with poor prognosis, where higher intermediate monocyte levels were linked to the severity of septic shock (Hortová-Kohoutková et al. 2020; Liepelt et al. 2020).

The generation of SARS-CoV-2-specific effector T cells is crucial for an effective adaptive immune response and long-term protection against the virus. Our findings show that intranasally infected *Rosa26*^*creERT2/chAce2*^ mice produce effector cytotoxic T cells more efficiently than intratracheally infected mice, which generated effector cytotoxic T cells without excessive pathology, suggesting that this route should be further explored for its potential to induce long-term protection against reinfection. As a future direction, exploring the infection of Rosa26^creERT2/chAce2^ mice with other SARS-CoV-2 variants remains a key area of interest. This approach could be particularly valuable in studying reinfections with different variants, better replicating the human scenario of sequential exposures and immune responses. Additionally, an important open question is which regions of the brain support viral replication and where the virus can be detected. While this was beyond the scope of the current study, future research could provide valuable insights into the neurotropism of SARS-CoV-2 and its potential neurological effects. Altogether, this *Rosa26*^*creERT2/chAce2*^ model provides a valuable tool for studying the multisystemic aspects of SARS-CoV-2 infection and evaluating potential therapeutic interventions.

## Electronic supplementary material

Below is the link to the electronic supplementary material.

Supplementary Material 1

## Data Availability

No datasets were generated or analysed during the current study.
