# Supplementary material for: New mouse model for inducible hACE2 expression enables to dissect SARS-CoV-2 pathology beyond the respiratory system
Source: Mamm Genome. 2025 Feb 22;36(2):403–16. doi: 10.1007/s00335-025-10115-1 (PMC12130128; doi:10.1007/s00335-025-10115-1)
Supplement: Supplementary file 1 — Supplementary Material 1 [file 335_2025_10115_MOESM1_ESM.docx]

**Online resource**

**Title:**

**New mouse model for inducible hACE2 expression enables to dissect SARS-CoV-2 pathology beyond the respiratory system.**

Federica Gambini^1^, Dominik Arbon^2^, Petr Nickl^2^, Vaclav Zatecka^2^, Olha Fedosieieva^2^, Juraj Labaj^2^, Vendula Novosadova^2^, Jana Trylcova^3^, Jan Weber^3^, Jan Prochazka^1,2^, Jana Balounova^2#^, Radislav Sedlacek^1,2#^

**Affiliations**

^1^Laboratory of Transgenic Models of Diseases, Institute of Molecular Genetics of the Czech Academy of Sciences, Videnska 1083, 142 20 Prague, Czech Republic

^2^Czech Centre of Phenogenomics, Institute of Molecular Genetics of the Czech Academy of Sciences, Prumyslova 595, 252 50 Vestec, Czech Republic

^3^Institute of Organic Chemistry and Biochemistry of the Czech Academy of Sciences, 166 10 Prague, Czech Republic

#Corresponding authors: [radislav.sedlacek@img.cas.cz](mailto:radislav.sedlacek@img.cas.cz); [jana.balounova@img.cas.cz](mailto:jana.balounova@img.cas.cz)

| **Specificity** | **Fluorochrome** | **Clone** | **Cat. no.** | **Manufacturer** | **Dilution** |
| --- | --- | --- | --- | --- | --- |
| CD8a | BUV395 | 53-6.7 | 563786 | BD Biosciences | 1:200 |
| CD44 | BUV805 | IM7 | 741921 | BD Biosciences | 1:400 |
| CD5 | BV421 | 53-7.3 | 562739 | BD Biosciences | 1:400 |
| CD11b | BV510 | M1/70 | 562950 | BD Biosciences | 1:1000 |
| Bst2 | BV605 | 927 | 127025 | Biolegend | 1:600 |
| GITR | BV711 | DTA-1 | 563390 | BD Biosciences | 1:400 |
| Ly6G | BV785 | 1A8 | 127645 | Biolegend | 1:300 |
| CD45 | FITC | 30-F11 | 103108 | Biolegend | 1:1000 |
| CD43 | RB705 | S7 | 757824 | BD Biosciences | 1:1500 |
| CD161 | PE | PK136 | 108707 | Biolegend | 1:800 |
| CD19 | PE-CF594 | 1D3 | 562329 | BD Biosciences | 1:800 |
| CD25 | PE-Cy7 | PC61 | 552880 | BD Biosciences | 1:400 |
| CD4 | APC | GK1.5 | 100411 | Biolegend | 1:1000 |
| Ly6C | AF700 | AL-21 | 561237 | BD Biosciences | 1:300 |
| CD62L | APC-Cy7 | MEL-14 | 560514 | BD Biosciences | 1:1500 |

***Online resource 1. The list of antibodies used for peripheral blood white blood cell analysis.***

**
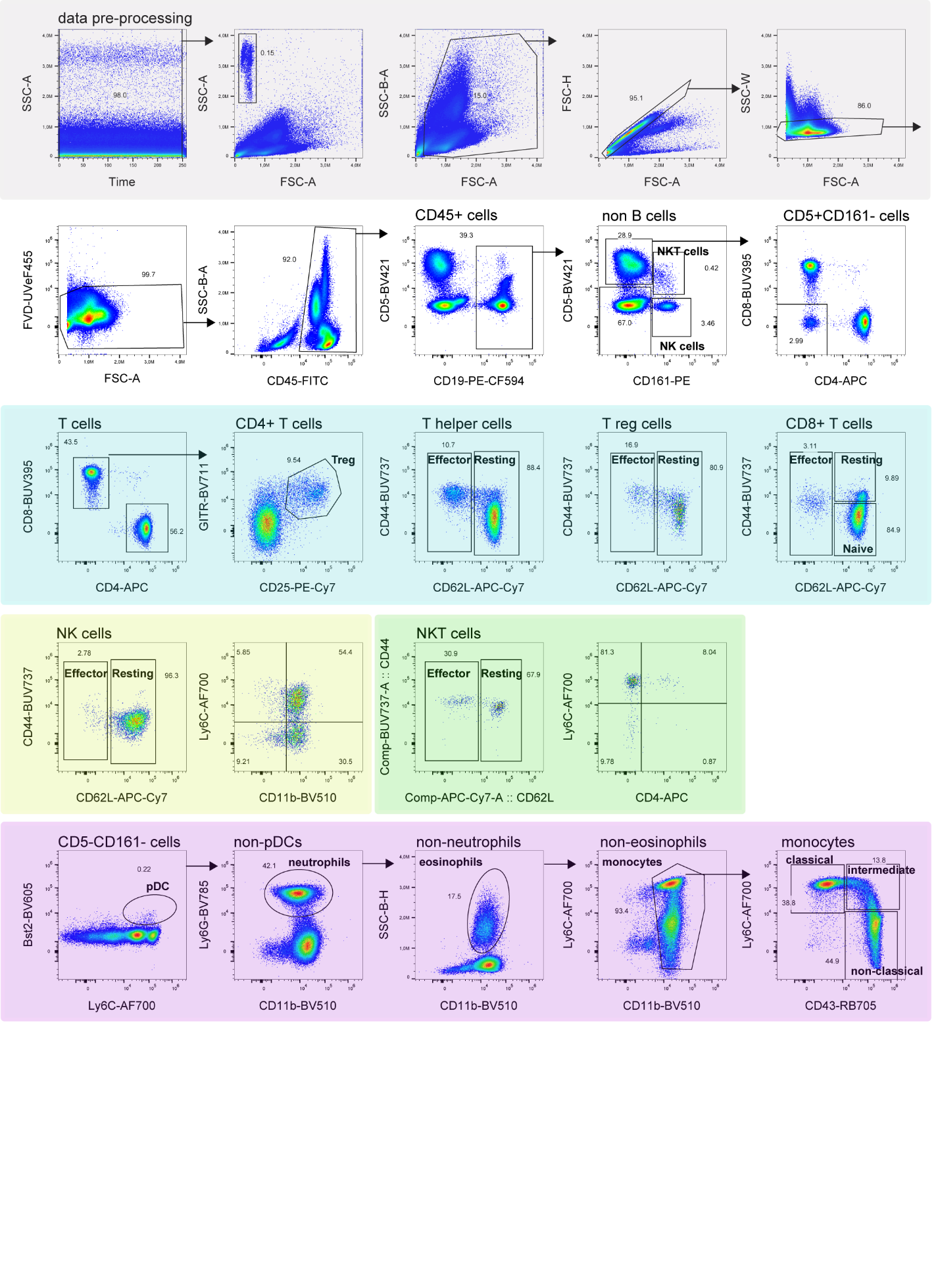
*Online resource 2. Gating strategy for flow cytometry analysis of peripheral blood white blood cells.***

**
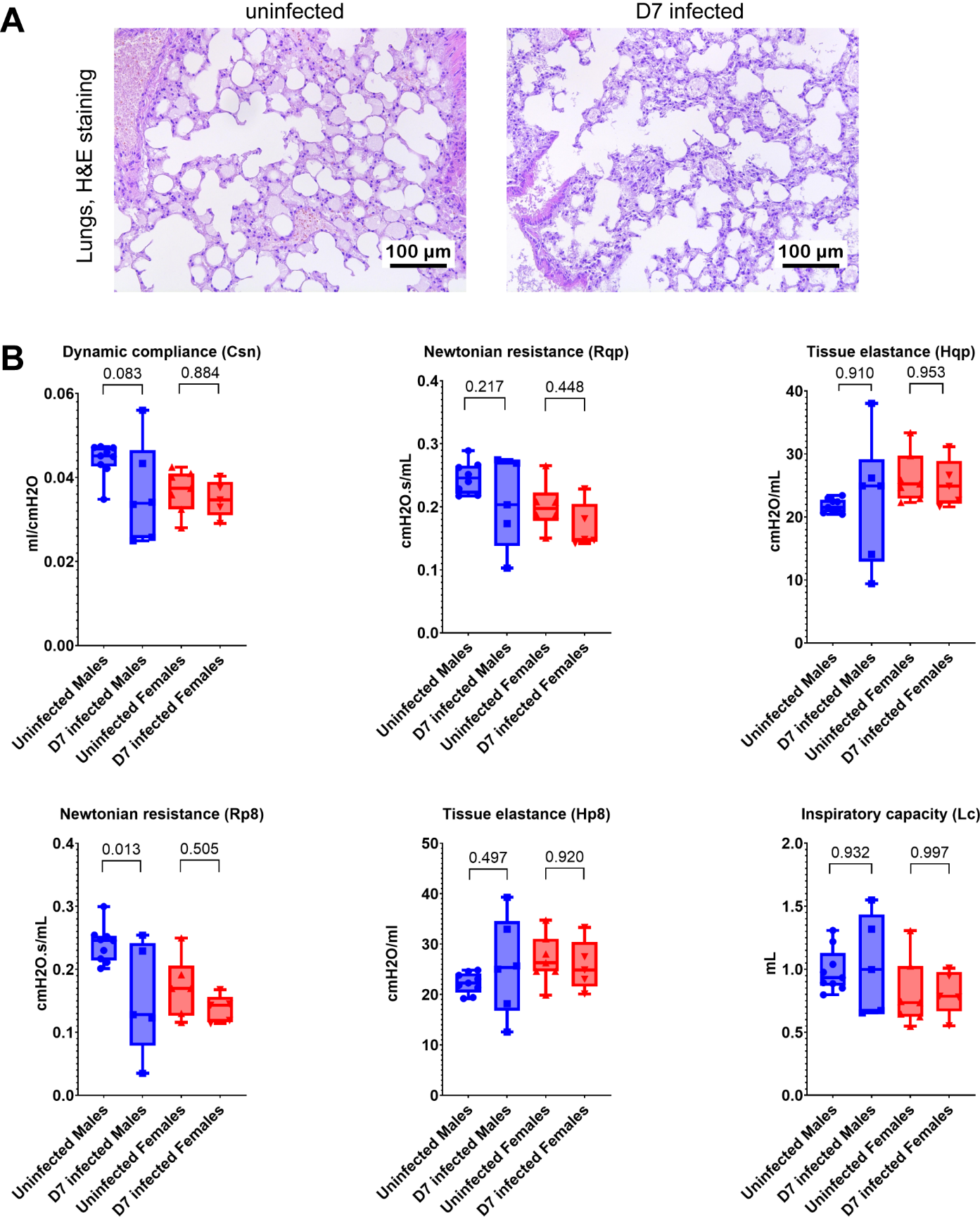
**

**Online Resource 3. FlexiVent parameters comparing uninfected and day 7 post-infection Rosa26^creERT2/chACE2^mice**. Dynamic compliance (Csn), Newtonian resistance (Rqp, Rp8), Tissue elastance (Hqp, Hp8), Inspiratory capacity (Lc). One-way ANOVA p-values are depicted above the graphs.

**Online Resource 4. The dynamics of B and Treg cells during infection in Rosa26^creERT2/chACE2^mice**
During the infection in Rosa26^creERT2/chACE2^ mice, B cells show a decline over time. A similar trend is observed in both Treg cells and effector Treg cells. Data are shown as mean ± SD For intranasal infection: Uninfected (n=11 males, n=12 females), D2 (n=4 males, n=4 females), D5 (n=4 males, n=3 females), D7 (n=3 males, n=5 females). For intratracheal infection: Uninfected (n=15 males, n=12 females), D2 (n=4 males, n=3 females), D5 (n=4 males, n=4 females), D7 (n=2 males, n=4 females).

**Online Resource 5. Monocytes shift from the classical to non-classical phenotype during infection in Rosa26^creERT2/chACE2^ mice.** Monocyte subsets were identified based on their expression of Ly6C and CD43 (Meghraoui-Kheddar, Barthelemy et al. 2020). Data are shown as mean ± SD For intranasal infection: Uninfected (n=11 males, n=12 females), D2 (n=4 males, n=4 females), D5 (n=4 males, n=3 females), D7 (n=3 males, n=5 females). For intratracheal infection: Uninfected (n=15 males, n=12 females), D2 (n=4 males, n=3 females), D5 (n=4 males, n=4 females), D7 (n=2 males, n=4 females).
